# Proximal Knee Ganglion Cyst: A Case Report and Review of Treatment Strategies

**DOI:** 10.7759/cureus.78651

**Published:** 2025-02-06

**Authors:** Yvana Toh

**Affiliations:** 1 Surgery, Surgical, Treatment and Rehabilitation Service (STARS) Queensland Health, Brisbane, AUS

**Keywords:** corticosteroid injection, knee ganglion cyst, knee joint soft tissue, knee mri, us-guided fine-needle aspiration

## Abstract

Ganglion cysts are commonly encountered benign lesions, primarily localized near the wrist and hand but occasionally observed in the lower extremities. These cysts, characterized by their encapsulation in dense connective tissue, typically present with minimal symptoms, albeit occasionally causing discomfort or limited mobility. Herein, a unique case of a ganglion cyst in the posterior distal lower extremity is presented, located near the popliteal neurovascular structures and without any connection to the knee joint. Given the critical proximity to neurovascular structures and the patient's mild symptoms, a non-surgical approach was favored. Ultrasound-guided aspiration coupled with cortisone injection yielded effective symptom relief, underscoring the efficacy of conservative management. This case underscores the distinctive extra-articular location of ganglion cysts and the importance of a thorough risk-benefit assessment in determining the optimal treatment strategy.

## Introduction

Ganglion cysts are benign formations encapsulated by dense connective tissue and filled with gelatinous fluid containing high levels of hyaluronic acid and mucopolysaccharides [1]. They are not lined by synovium, may be unilocular or multilocular and often have internal septa. While the precise etiology of ganglion cyst development remains elusive, it is theorized to stem from repetitive microtrauma-inducing mucinous degeneration of connective tissue. This process is thought to be mediated by fibroblasts stimulated by repetitive injury to capsular and ligamentous structures, resulting in the accumulation of hyaluronic acid and the formation of the characteristic mucin substance within ganglion cysts [2-4].

Most ganglion cysts are asymptomatic but can occasionally present with pain, swelling, and discomfort around the knee [5]. Treatment strategies encompass both non-operative and surgical modalities, although a notable recurrence rate has historically hindered the efficacy of non-surgical interventions. Surgical excision remains a definitive treatment option for symptomatic cases; however, carefully considering the adjacent anatomical structures is paramount to mitigate the risk of inadvertent injury to nearby neurovascular elements [6].

## Case presentation

A 40-year-old male, without any known underlying medical conditions, was referred to our orthopedic outpatient clinic by his local general practitioner due to vague left-sided knee discomfort that had been intermittently occurring for the past two years. He reported experiencing sharp pain when flexing his knee, particularly while bearing weight at a 30-degree angle. However, the pain subsided when the knee was fully extended or flexed beyond this angle. Despite these symptoms, he maintained full weight-bearing capacity and could engage in high-impact activities like football, as well as manage his physically demanding job as an aircraft engineer, which involves extensive walking. Upon examination, no abnormalities were detected in the patient's knee. Despite efforts, we were unable to reproduce the reported pain during the consultation.

**Figure 1:**
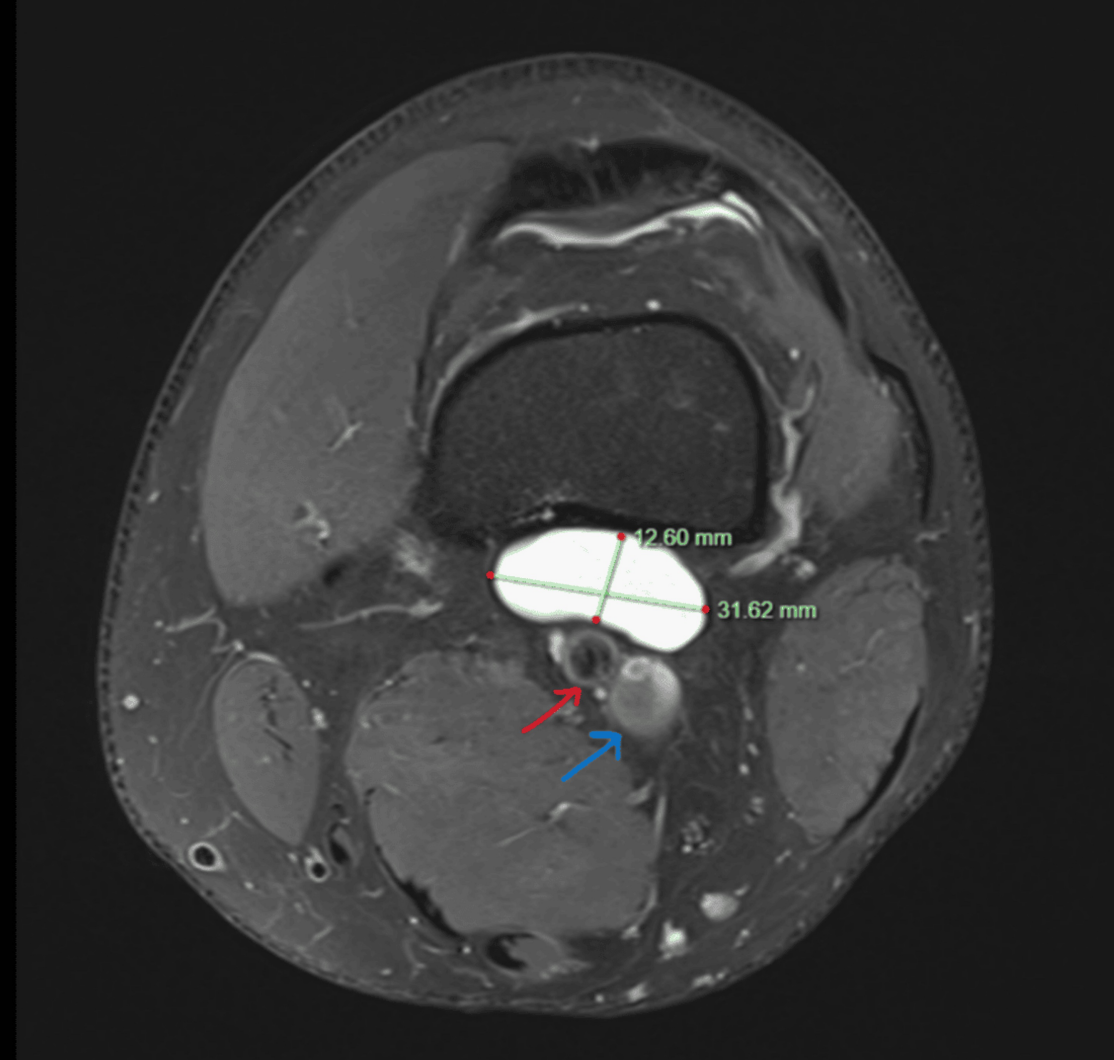
T2-weighted MRI of the left knee (axial view) shows a well-defined, ovoid cystic structure measuring 31.62 × 12.60 mm, abutting the posterior cortex of the distal femur and positioned anterior to the popliteal artery (red arrow) and popliteal vein (blue arrow).

**Figure 2:**
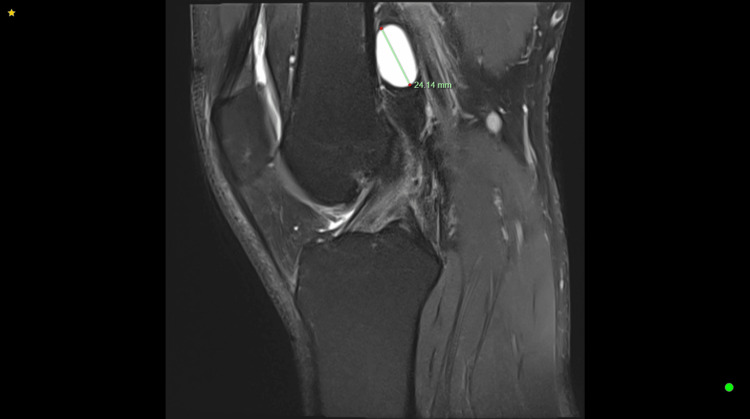
T2-weighted MRI of the left knee (sagittal view) shows a well-defined ovoid cystic structure measuring 24.14 mm, abutting the posterior cortex of the femur.

A non-contrast MRI of the left knee revealed a well-defined, sizable cystic structure measuring 31.62 × 12.60 × 24.14 mm, located adjacent to the posterior cortex of the distal femur, anterior to the popliteal artery, vein, and nerve, with no evidence of intra-articular pathology. Considering its proximity to the neurovascular bundle, non-surgical management was deemed preferable to surgical excision. Consequently, we performed ultrasound-guided aspiration and fenestration of the cystic structure, followed by an injection of hydrocortisone (1 mL Celestone mixed with 1% lignocaine) into the cyst space. The patient was encouraged to monitor his symptoms and keep a pain diary from the point of injection till his review appointment four months later. The patient reported complete resolution of his symptoms during his follow-up appointment. A histology report for the aspirated fluid in the knee joint confirmed our suspicion of a ganglion cyst. This approach successfully alleviated the patient's symptoms without the need for surgical intervention (Figures 1, 2).

## Discussion

Ganglion cysts are benign, fluid-filled lesions typically found near joints or tendons. They are most commonly found on the wrist or hand but are also frequently encountered in the knee and foot [6]. Standard MRI imaging typically reveals cystic characteristics, displaying low signal intensity on T1-weighted imaging (T1WI) and high signal intensity on T2-weighted imaging (T2WI). However, if complicated by hemorrhage or infection, they may exhibit heterogeneous features, often presenting with irregularly delineated and show pericapsular edema without enhancing soft tissue components upon contrast administration [7]. These cysts can manifest intra-articularly, extra-articularly, intraosseously, or periosteally and may occur in various locations around the knee joint. Differential diagnoses for ganglion cysts include synovial cysts. These commonly have a synovial lining, and although they are histologically distinct from ganglia, they are indistinguishable on imaging [8]. 

Treatment options encompass both non-surgical and surgical approaches. Critical considerations include the proximity of surrounding anatomical structures, severity of symptoms, and recurrence risk. Non-surgical interventions may be attempted based on cyst location, with aspiration being a common method to alleviate symptoms [6]. Imaging-guided aspiration followed by cortisone injection may provide symptom relief and slow down recurrence. A study conducted by Grégoire and Guigal shows a 73.2% recurrence of a ganglion cyst on the wrist treated with corticosteroid injections [9].

Surgical intervention is warranted for patients with persistent symptoms unresponsive to conservative management. However, inadequate resection of the ganglion cyst pedicle, capsular attachments, and capsule remnants has been linked to high recurrence rates. Studies report recurrence rates of around 10% following excision of lower extremity ganglia [10,11]. Hence, a thorough evaluation of the risks and benefits is crucial before opting for surgical intervention. Despite the potential for complete eradication with surgery, given the proximity to the popliteal vasculature and minimal symptoms in our case, non-surgical management is deemed the optimal approach.

## Conclusions

In conclusion, ganglion cysts, characterized by benign encapsulation and gelatinous fluid content, pose diagnostic and therapeutic challenges, particularly when located in close proximity to vital anatomical structures. Non-surgical interventions, including aspiration and cortisone injection, offer symptomatic relief but may be associated with high recurrence rates. Surgical excision, although effective, necessitates meticulous attention to prevent recurrence and mitigate risks to adjacent neurovascular elements. In our case, a non-surgical approach was preferred due to the cyst's proximity to critical neurovascular structures and the patient's minimal symptoms, with ultrasound-guided aspiration and cortisone injection effectively relieving discomfort, underscoring the importance of considering individual patient factors and risk-benefit assessments in determining the optimal treatment strategy.
